# Study of 32 new phage tail-like bacteriocins (pyocins) from a clinical collection of *Pseudomonas aeruginosa* and of their potential use as typing markers and antimicrobial agents

**DOI:** 10.1038/s41598-022-27341-1

**Published:** 2023-01-03

**Authors:** Lucía Blasco, Manuel González de Aledo, Concha Ortiz-Cartagena, Inés Blériot, Olga Pacios, María López, Laura Fernández-García, Antonio Barrio-Pujante, Marta Hernández-Garcia, Rafael Cantón, María Tomás

**Affiliations:** 1 Microbiología Traslacional y Multidisciplinar (MicroTM)-Instituto de Investigación Biomédica (INIBIC), Servicio de Microbiología, Hospital A Coruña (CHUAC), Universidad de A Coruña (UDC), A Coruña, Spain; 2 Study Group on Mechanisms of Action and Resistance to Antimicrobials (GEMARA) the Behalf of the Spanish Society of Infectious Diseases and Clinical Microbiology (SEIMC), Madrid, Spain; 3 Servicio de Microbiología, Hospital Universitario Ramón y Cajal, Instituto Ramón y Cajal de Investigación Sanitaria (IRYCIS), CIBER de Enfermedades Infecciosas (CIBERINFEC), Instituto de Salud Carlos III, Madrid, Spain

**Keywords:** Molecular medicine, Bacterial genetics

## Abstract

Phage tail-like bacteriocins (PTLBs) are large proteomic structures similar to the tail phages. These structures function in bacterial competition by making pores in the membrane of their competitors. The PTLBs identified in *Pseudomonas aeruginosa* are known as R-type and F-type pyocins, which have a narrow spectrum of action. Their specificity is determined by the tail fiber and is closely related to the lipopolysaccharide type of the target competitor strain. In this study, the genome sequences of 32 clinical of *P. aeruginosa* clinical isolates were analysed to investigate the presence of R-type and F-type pyocins, and one was detected in all strains tested. The pyocins were classified into 4 groups on the basis of the tail fiber and also the homology, phylogeny and structure of the cluster components. A relationship was established between these groups and the sequence type and serotype of the strain of origin and finally the killing spectrum of the representative pyocins was determined showing a variable range of activity between 0 and 37.5%. The findings showed that these pyocins could potentially be used for typing of *P. aeruginosa* clinical isolates, on the basis of their genomic sequence and cluster structure, and also as antimicrobial agents.

## Introduction

*Pseudomonas aeruginosa* is a Gram-negative opportunistic pathogen, responsible for nosocomial infections, including bloodstream infections, pneumonia, urinary tract infections and surgical site infections^1^. This pathogen represents a serious problem in health care systems because of its capacity to acquire antibiotic resistance and its ability to produce biofilms and persist on surfaces, thereby contributing to its spread and causing outbreaks^2^. *P. aeruginosa* contains many intrinsic antibiotic resistance mechanisms that make this species a difficult to treat multidrug resistant bacteria^3^. In addition, due to the outbreaks, it is necessary to type the causative bacterial pathogen. The traditional typing methods, Pulse Field Gel Electrophoresis (PFGE) and the Multilocus Sequencing Typing (MLST), are the primary election in many clinical laboratories, but although they are very effective it should be necessary to improve the discrimination inside each Sequence Type (ST)^4^. So, new antimicrobial agents as well as new typing methods are therefore required.

Bacteria utilize phage tail-like bacteriocins (PTLBs) to enable them to compete with other strains of the same species or with different species^5^ and could therefore be good candidates for use as antimicrobial agents. PTLBs are large, ribosomally synthetized^6^ protein structures (2 × 10^6^–1 × 10^7^ KDa) encoded in the bacterial genome and structured in genetic clusters similar to the phage tail structure models, always encoding structural and assembly tail proteins, a lysis cassette and preceded by the transcription regulatory proteins^5^.

*P. aeruginosa* produces PTLBs called pyocins, traditionally named after the species of origin, in this case *P. aeruginosa* also known as *P. pyocyanea*^5^. The PTBLs from *P. aeruginosa* are some of the most widely studied PTLBs and are used as PTLBs models^5^. The *P. aeruginosa* pyocins are grouped into three types: S-type pyocins, R-type pyocins and F-type pyocins. The S-type pyocins, not considered PTLBs, are colicin-like proteins composed by large multi domain polypeptides with DNase activity^7^. The R-type pyocins and F-type pyocins are considered PTLBs and differ in their structure. R-type (Rigid-type) pyocins are contractile tail particles similar to the *Myoviridae* phage family and are composed by a long large tube surrounded by a sheath and ending in a baseplate where the receptor-binding protein (RBP) is located. The F-type (Flexible-type) pyocins are non-contractile particles, similar to the *Siphoviridae* phage family; they are simpler than the R-type as they do not possess a sheath, but also have an RBP^5,8^. Despite the great morphological similarity of PTBLs with phages, they cannot be considered degenerate phages but both would have a common cellular ancestry along with the type VI secretion system^5,8,9^.

The killing mechanism of R-type and F-type pyocins involves the phage tail fiber possessing the RBP, which recognizes the bacterial receptor in the lipopolysaccharide (LPS). The specificity of the RBP leads to a narrow spectrum of activity and the protection of the pyocin producer strain, although the producer cell dies altruistically due to the pyocins release^5^; once the cell is bound, the R-type pyocin triggers contraction of the sheath by pulling the tail and the core through the envelope, forming a channel or pore that decouples the membrane potential thus inhibiting membrane transport and causing cell death^3,5^. The killing mechanism of the F-type also involves disruption of the membrane gradient, but as it lacks a tube or core that forms a channel, its mechanism of action is not yet well understood^5,10^. The S-type pyocins are secreted as binary protein complexes that contain an effector, which is a larger protein with killing activity including DNase activity, the second component is a smaller protein with immunity activity that protects the producer strain from the activity of the released pyocin^11,12^.

To date, five subtypes of R-type pyocins have been described on the basis of their target specificity: R1, R2, R3, R4 and R5^8,12,13^. Of these, R5 has the broadest spectrum, while R2 has the narrowest spectrum and encompasses the spectra of R3 and R4, which are similar. R1 is a subset of R5 but is considered a different branch. In addition, the amino acid sequence of the tail fiber protein is similar in R2, R3 and R4, but very different in R1 and R5^5,13,14^. The F-type pyocins have been less well studied and are currently classified into three groups (F1, F2 and F3) based on their lytic spectra, although another two groups have also been identified: one in *P. aeruginosa* PA14 and other in *P. aeruginosa* M18. The former is similar to F2, identified in the reference strain *P. aeruginosa* PAO1, but with differences in 210 to 340 residues from the tail fiber protein; the M18 F-type shares 66% homology with the tail fiber protein of the F1 group^12^.

PTBLs are encoded in gene clusters preceded by a group of regulatory genes and organised in blocks of lytic and structural genes. These clusters can be found encoding individual R-type or F-type pyocins but also in dual R-F pyocin clusters. It has previously been reported that all clusters in *P. aeruginosa*, i.e. both individual and the dual R-F type, are located in the genome in the intergenic region of the tryptophan operon between the *trpE* and *trpGCD* genes^5,12^. The regulatory system of both individual and dual R-type and F-type pyocins is located upstream of the cluster and is composed by an activator (*prtN*) and a repressor (*prtR*); these are related to *recA*, which is activated by DNA damage, cleaving *prtR* and producing *prtN* and thus activating expression of the pyocin cluster^5,12,15^. The production of both R-type and F-type pyocins in dual R-F pyocins relies on the shared regulatory system and also the shared lysis cassette, suggesting coordinated release of the dual pyocins^8,12^.

PTLBs are characterized by a narrow host spectrum, which in *P. aeruginosa* is known to be closely related to the LPS type of the target strain that acts as a receptor for the tail fiber proteins^14^. It has also been reported that each specific type of pyocin recognizes a specific serotype strain determined by the O-polysaccharide repeating portion, which acts as an O-antigen and is linked to the virulence of the strains^14,16^. The PTLBs, and more specifically the *P. aeruginosa* pyocins, can be used as antimicrobial agents and owing to their relationship with the O-antigen they were used in typing schemes before the emergence of molecular typing methods^17–19^. The value of pyocins as antimicrobial agents is currently being considered in research studies. The R-type pyocins were used for the first time in 1969 to rescue chick embryos infected with *P. aeruginosa*^20^. In addition, several assays in murine models confirmed the efficacy of R-type pyocins in the treatment of infections with *P. aeruginosa*^13,21,22^. Due to the narrow spectrum of the PTLBs, some studies focused on generating recombinant R-type pyocins to retarget them by substituting the tail fiber protein with a phage protein in order to increase the host range in several species such as *P. aeruginosa*, *Clostridioides difficile* and *Listeria monocytogenes*^10,23–26^.

In the search for new pyocins, 32 genome sequences of clinical strains of *P. aeruginosa* of different clinical origins were analysed in the present study, and at least one pyocin cluster was identified in each strain. The sequence of these pyocins was analysed and homology and phylogenetic studies were conducted. The pyocins were isolated and purified and their killing spectrum was established, and they were also related to the serotype and ST of the clinical isolates.

## Results

### Identification and characterization of the phage tail-like bacteriocins

Thirty-two genomic sequences of *P. aeruginosa* were analysed to search for PTLBs (Table 1). In all of the genomes analysed, at least one cluster corresponding to a pyocin was found between tryptophan operon genes, *trpE* and *trpG* (Table 2; Table 1S. Supplementary material). Thus, 21 of the strains contained a cluster that corresponded to a unique pyocin corresponding to an R-type pyocin. Dual clusters were identified in the 11 remaining strains. In the *P. aeruginosa* PAO1 reference strain^5^, one of these clusters corresponded to a R-type pyocin, and the contiguous cluster corresponded to an F-type pyocin, both sharing the regulatory and lytic genes, as previously described for *P. aeruginosa* PAO1^12^.

**Table 1:** Characteristics of the 32 clinical isolates of *P. aeruginosa* which were obtained in previous study^30^.

Strain	Genbank	Origin	ST	Serotype
1-13	SAMN14776823	IAI	ST235	O11
2-29	SAMN14776826	UTI	ST235	O11
3-49	SAMN14776829	IAI	ST235	O11
4-17	SAMN14776829	UTI	ST235	O11
4-71	SAMN14776839	IAI	ST235	O11
4-79	SAMN14776840	UTI	ST235	O11
4-86	SAMN14776841	IAI	ST235	O11
4-92	SAMN14776842	UTI	ST235	O11
4-93	SAMN14776843	UTI	ST235	O11
4-94	SAMN14776844	IAI	ST235	O11
4-120	SAMN14776833	UTI	ST235	O11
4-121	SAMN14776834	UTI	ST235	O11
9-41	SAMN14776860	LRTI	ST235-1LV	O11
C11	SAMN14776862	UTI	ST175	O4
C58	SAMN14776863	UTI	ST175-2LV	O4
G6	SAMN14776870	IAI	ST175-1LV	O4
G7	SAMN14776871	IAI	ST175-2LV	O4
G26	SAMN14776868	IAI	ST175-1LV	O4
G31	SAMN14776869	IAI	ST175-1LV	O4
H18	SAMN14776872	UTI	ST175-2LV	O4
H52	SAMN14776874	UTI	ST309	O11
3-5	SAMN14776830	UTI	ST348-1LV	O11
3-38	SAMN14776827	LRTI	ST348	O12
3-41	SAMN14776828	LRTI	ST348-1LV	O12
3-58	SAMN14776831	IAI	ST348	O11
9-86	SAMN14776861	IAI	ST554	O5
5-23	SAMN14776846	LRTI	ST244	O5
6-25	SAMN14776848	LRTI	ST244-1LV	O12
8-24	SAMN14776854	UTI	ST244-1LV	O5
8-36	SAMN14776855	UTI	ST244	O5
9-25	SAMN14776858	LRTI	ST244-1LV	O12
10-58	SAMN14776820	LRTI	ST244	O12

It is indicated each Genbank code, clinical origin, sequence type (ST) and serotype.

*IAI* intra-abdominal infection, *UTI* urinary tract infection, *LRTI* lower respiratory tract infection.

**Table 2:** Genomic characteristics and subtype of the 32 pyocins (PTBLs) identified in this study.

Pyocin name	Pyocin subtype	Genbank	Genomic lenght (pb)	% GC	ORF	Group
1-13_pyoR5	R5	BK062625	16,471	65.4	23	**A**
2-29_pyoR5	R5	BK062626	16,471	65.4	23	
3-49_pyoR5	R5	BK062627	16,471	65.4	23	
4-17_pyoR5	R5	BK062628	16,472	65.5	23	
4-71_pyoR5	R5	BK062629	16,471	65.4	23	
4-79_pyoR5	R5	BK062630	16,471	65.4	23	
4-86_pyoR5	R5	BK062631	16,471	65.4	23	
4-92_pyoR5	R5	BK062632	16,471	65.4	23	
4-93_pyoR5	R5	BK062633	16,471	65.4	23	
4-94_pyoR5	R5	BK062634	16,803	65.4	23	
4-120_pyoR5	R5	BK062635	16,471	65.4	23	
4-121_pyoR5	R5	BK062636	16,803	65.4	23	
9-41_pyoR5	R5	BK062645	16,803	65.3	23	
C11_pyoR5	R5	BK062637	16,455	65.1	23	**B**
C58_pyoR5	R5	BK062638	16,455	65.1	23	
G6_pyoR5	R5	BK062639	16,312	65	23	
G7_pyoR5	R5	BK062640	16,120	65.2	23	
G26_pyoR5	R5	BK062641	16,455	65.1	22	
G31_pyoR5	R5	BK062642	16,455	65.1	22	
H18_pyoR5	R5	BK062643	16,120	65.2	22	
H52_pyoR5	R5	BK062644	16,215	65.3	22	
3-5_pyoR2-F2	R2-F2	BK062615	30,282	63.9	39	**C**
3-38_pyoR2-F2	R2-F2	BK062614	30,282	63.9	39	
3-41_pyoR2-F2	R2-F2	BK062616	30,282	63.9	39	
3-58_pyoR2-F2	R2-F2	BK062617	30,283	63.9	39	
9-86_pyoR2-F2	R2-F2	BK062622	30,510	64	41	
5-23_pyoR2-F(PA14)	R2-F(PA14)	BK062618	28,526	64.2	36	**D**
6-25_pyoR2-F(PA14)	R2-F(PA14)	BK062619	28,805	64.2	36	
8-24_pyoR2-F(PA14)	R2-F(PA14)	BK062620	28,805	64.2	36	
8-36_pyoR2-F(PA14)	R2-F(PA14)	BK062621	28,526	64.2	36	
9-25_pyoR2-F(PA14)	R2-F(PA14)	BK062623	28,805	64.2	36	
10-58_pyoR2-F(PA14)	R2-F(PA14)	BK062624	28,526	64.2	36	

Each pyocin name corresponds to the producer strain. It is indicated the Genbank code, the genome size, % GC, number of ORF and the group to which each was assigned.

**Figure 1:**
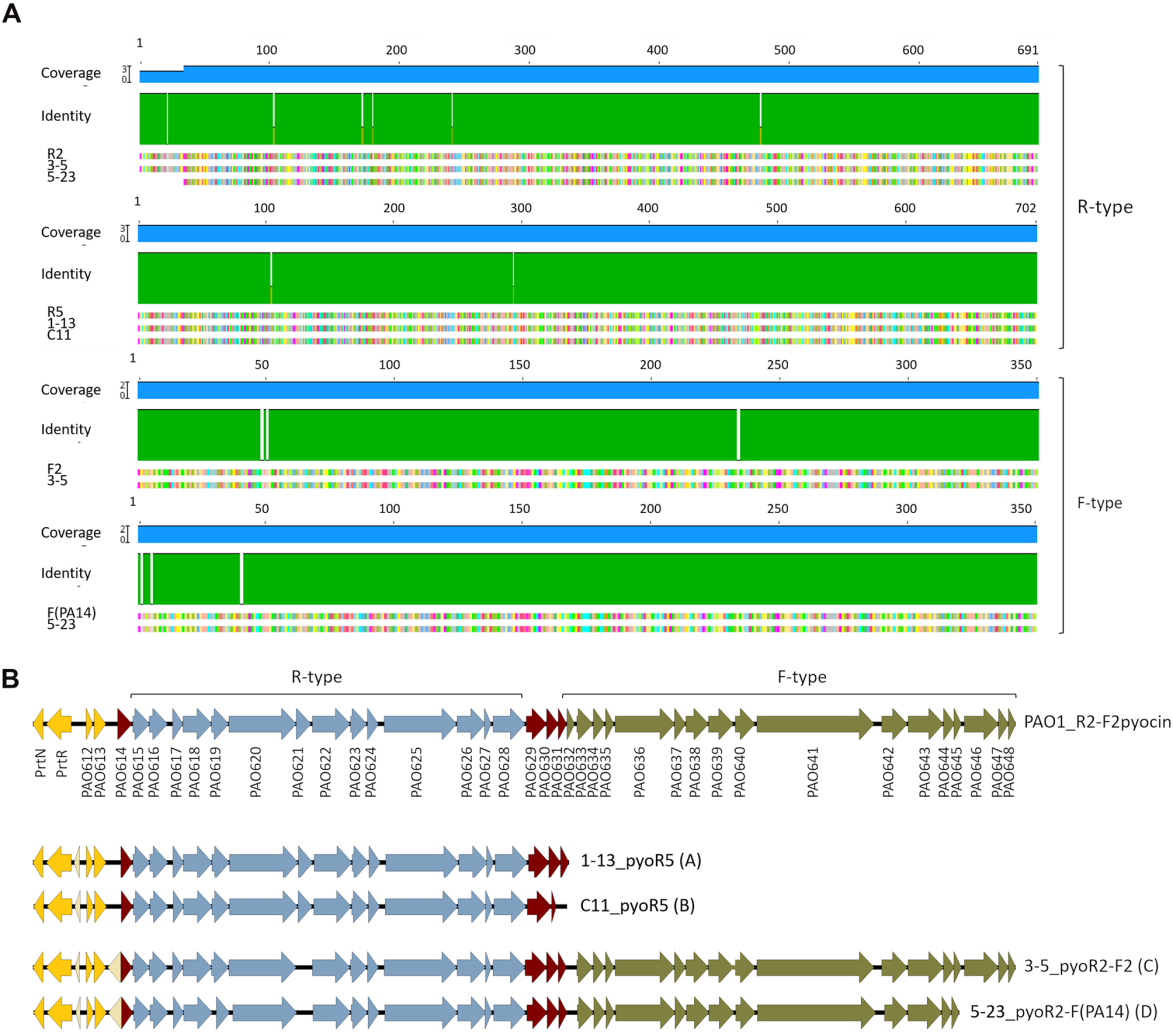
Identification of the pyocin subgroup. (**A**) Homology comparison of the tail fiber protein model sequence of R2, R5, F2 and F(PA14) with one representative of each pyocin type identified in this study. (**B**) Structure of the pyocin cluster of one representative of each group (A, B, C and D), and comparison with the reference pyocin R2-F2 from *P. aeruginosa* PAO1. Yellow: regulatory genes; red: lysis cassette; blue: R-type pyocin cluster genes, brown: F-type pyocin cluster genes. The representative sequences were selected by numerical order due to the high intragroup similarity.

In order to identify the PTLBs as R or F subtypes, homology analysis of the tail fiber was conducted. In the R-type pyocins, the tail fiber proteins were compared against the reference sequence for each R subtype (R1, R2, R3, R4 and R5); the results revealed that 21 of the proteins belonged to the R5 subtype, while 11 belonged to the R2 subtype, which corresponded to those that were followed by a F-type pyocin (Fig. 1A,B). For the F-type group, the results showed a group of 5 pyocins belonging to the F2 subtype, comprising a R2-F2 pyocin, similar to the PAO1 R2-F2 pyocin^5^, and a group of 6 that were similar to the *P. aeruginosa* PA14 F-type pyocin^12^, thus giving rise to a pyocin cluster R2-F(PA14) (Fig. 1A,B).

The genomic analysis of the pyocin clusters identified showed a % GC content very similar, between 63.9 and 65.4 (Table 2). Analysis of the protein sequence of the pyocins revealed some differences in the protein number between the R5-type pyocins (Table 2). Thus, these pyocins were classified in two groups, including a group of 12 pyocins (group A) constituted by an R-type cluster of 14 genes, 4 lytic genes and preceded by 5 regulator genes. The second group (group B) of 8 pyocins differed from group A in the absence of the latter protein belonging to the lytic cassette. In both groups, the cluster was preceded by the regulatory region composed by 5 genes, while in the reference *P. aeruginosa* PAO1 strain it is composed by 4 genes (Fig. 1B; Table 2)^7^. The R2-F2 pyocins (group C) were composed by 38 genes, which corresponded to an R-type cluster of 14 proteins and a F-type cluster composed by 16 genes, while in *P. aeruginosa* PAO1 the F-type cluster is formed by 17 genes. In addition, the two clusters share a regulatory region of five genes and a lytic cassette composed by four proteins (Fig. 1B). The pyocins R2-F(PA14) (group D) comprised two consecutive R and F clusters: the R-type comprised 14 proteins and the F-type comprised 13 proteins, unlike *P. aeruginosa* PAO1 and the group C pyocins, in which the last three proteins are duplicated (Fig. 1B)^7^.

### Homology and phylogenetic analysis of pyocins

**Figure 2:**
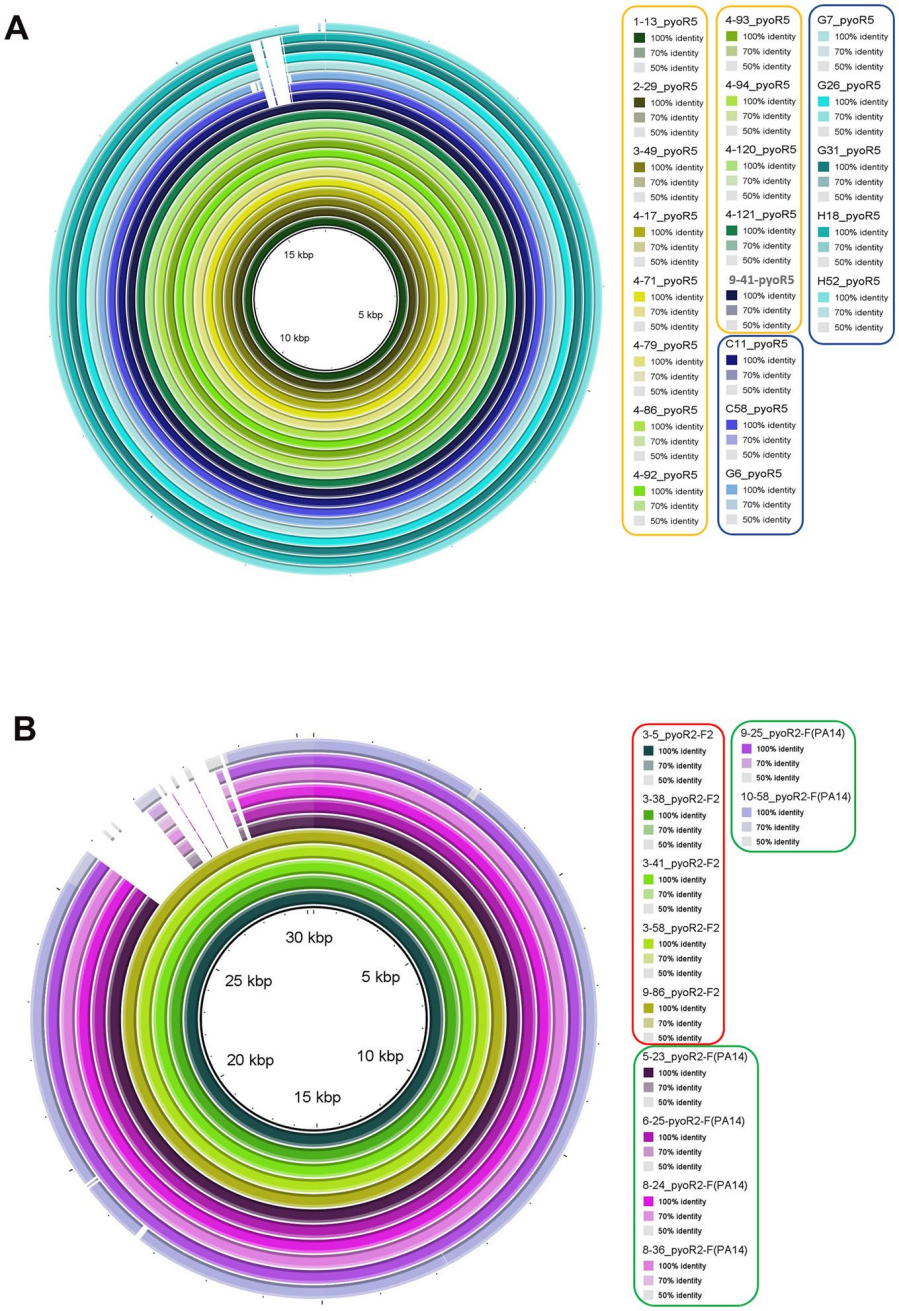
Homology of the pyocin clusters determined by BRIG 0.95. (**A**) R-type pyocin clusters. The clusters corresponding to group A are rounded in yellow and to group B in blue. (**B**) R-F type clusters. The cluster corresponding to group C are rounded in red and to group D in green.

The results obtained by the homology studies of the tail fiber specificity genes were confirmed by the homology and phylogenetic analysis of the complete pyocin genomes. The homology analysis showed that the R5-type pyocins were very similar and can be grouped in two blocks corresponding to the established groups A and B, sharing a query cover value of 97–98% and an identity value of 99.35%. The pyocin H52-R5 homology BRIG differed slightly from the two blocks but had similar homology values (Fig. 2). In the case of the R-F pyocin clusters, the homology results showed two blocks of homology, one corresponding to the R2-F2 pyocins (group C) and another corresponding to the R2-F(PA14) pyocin (group D) (Fig. 2). Despite the presence of two groups, the pyocin clusters represented by them were also similar, with a query cover value between 88 and 89% and an identity value of 98%.

**Figure 3:**
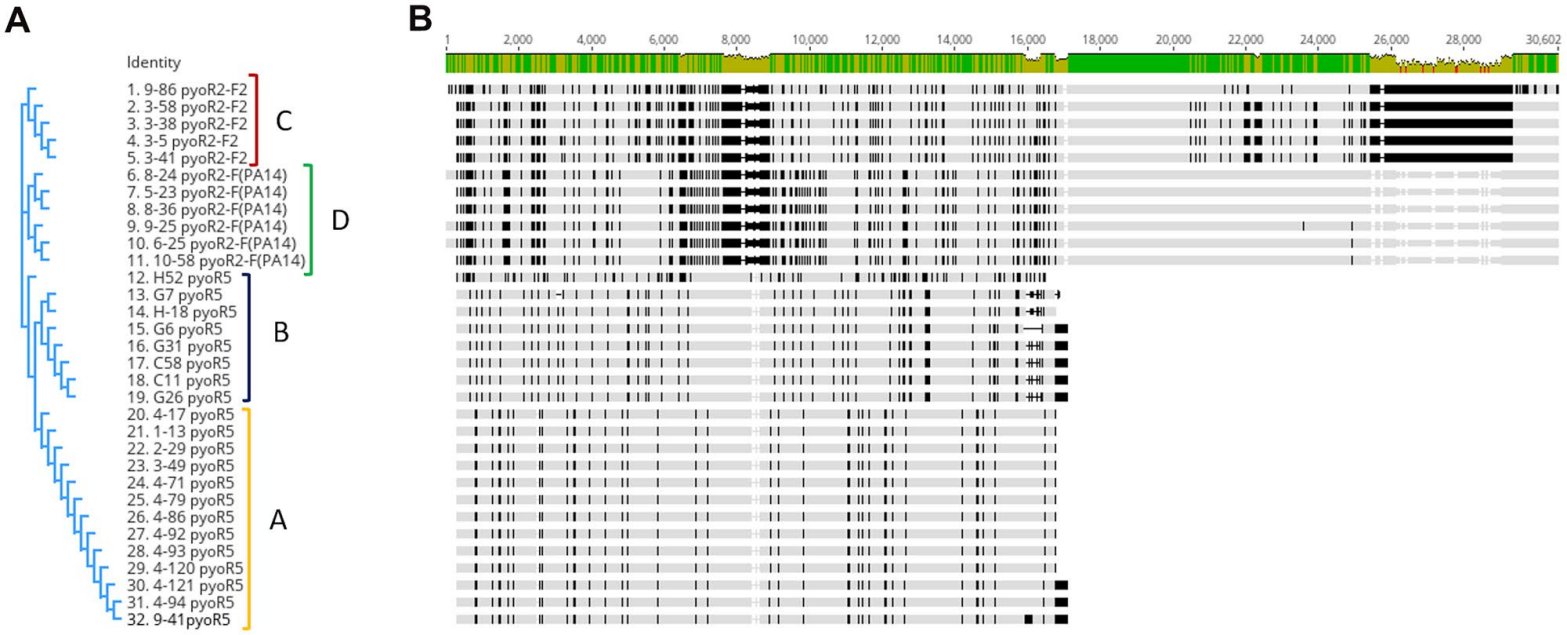
Similarity study of the 32 pyocin clusters identified and its relation with the established groups. (**A**) Phylogenetic tree. (**B**) Alignment of the 32 pyocins clusters.

The phylogenetic study of all the pyocin clusters revealed, as previously observed, that the pyocins identified are divided into four phylogenetic groups. Two closely related clades of the phylogenetic tree were represented by two blocks, one corresponding to the R5-type pyocins included in group A and another also corresponding to an R5-type pyocin grouped in B. Another two closely related clades included one corresponding to group C, which was constituted by the R2-F2 pyocin and group D, constituted by the R2-F(PA14) (Fig. 3).

### Identification of pyocins by transmission electron microscopy (TEM)

**Figure 4:**
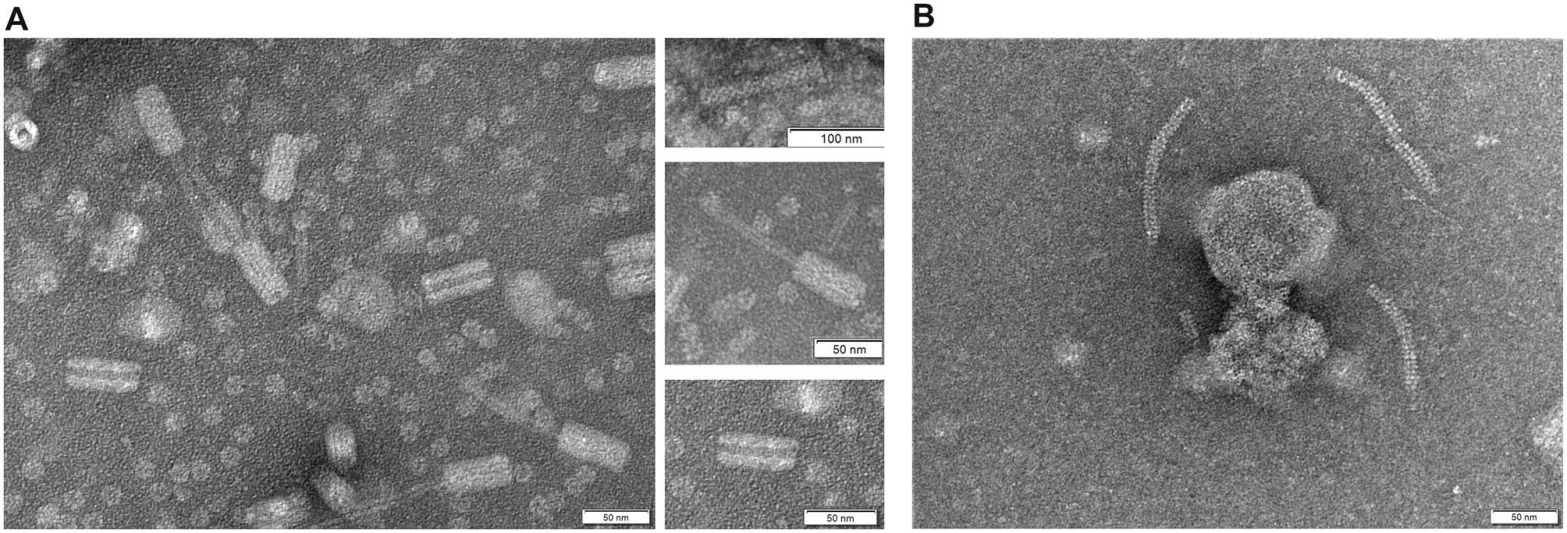
TEM images of pyocins. (**A**) R-type pyocin with a similar structure to a myovirus, in three conformations. Top: complete form; middle: contracted form; bottom: empty sheath of the pyocin. (**B**) F-type pyocin with a similar structure to a syphovirus.

The purified pyocins were examined by TEM, and the images obtained (Fig. 4) revealed the presence of two different type of pyocins. One type had a structure similar to a tail of the viral family Myoviridae, corresponding to the R-type pyocins, observed in three conformations: a complete form, a contracted form and an empty sheath^27^. The other type was the F-type, observed as a flexible structure similar to a phage tail of the viral family Syphoviridae.

### Relationship between the pyocin type and the source *P. aeruginosa* strain serotype, sequence type and clinical origin

In this analysis, the relationship between the serotype, the sequence type (ST) and the clinical origin of the source strains and the pyocin type was determined (Table 1). The R5-type pyocin in group A was almost always present in ST235 and serotype O11 clinical isolates. However, pyocin H52_pyoR5 was quite different in the homology and phylogenetic analysis, as it was present in the H52 clinical isolate belonging to the ST309 but like the rest of group A had the O11 serotype. The R5-type pyocins from group B were associated with clinical isolates belonging to the ST175 with serotype O4. On the other hand, the group C of R2-F2 pyocins was the most variable, with isolates belonging to the ST348 and ST554 and serotype O5, O11 and O12. Finally, group D, constituted by R2-F(PA14) pyocins, was represented by isolates belonging to ST244 and serotypes O5 and O12. Finally, no relationship between the clinical origin of the isolate and each pyocin was observed.

### Killing spectrum of the pyocins

**Figure 5:**
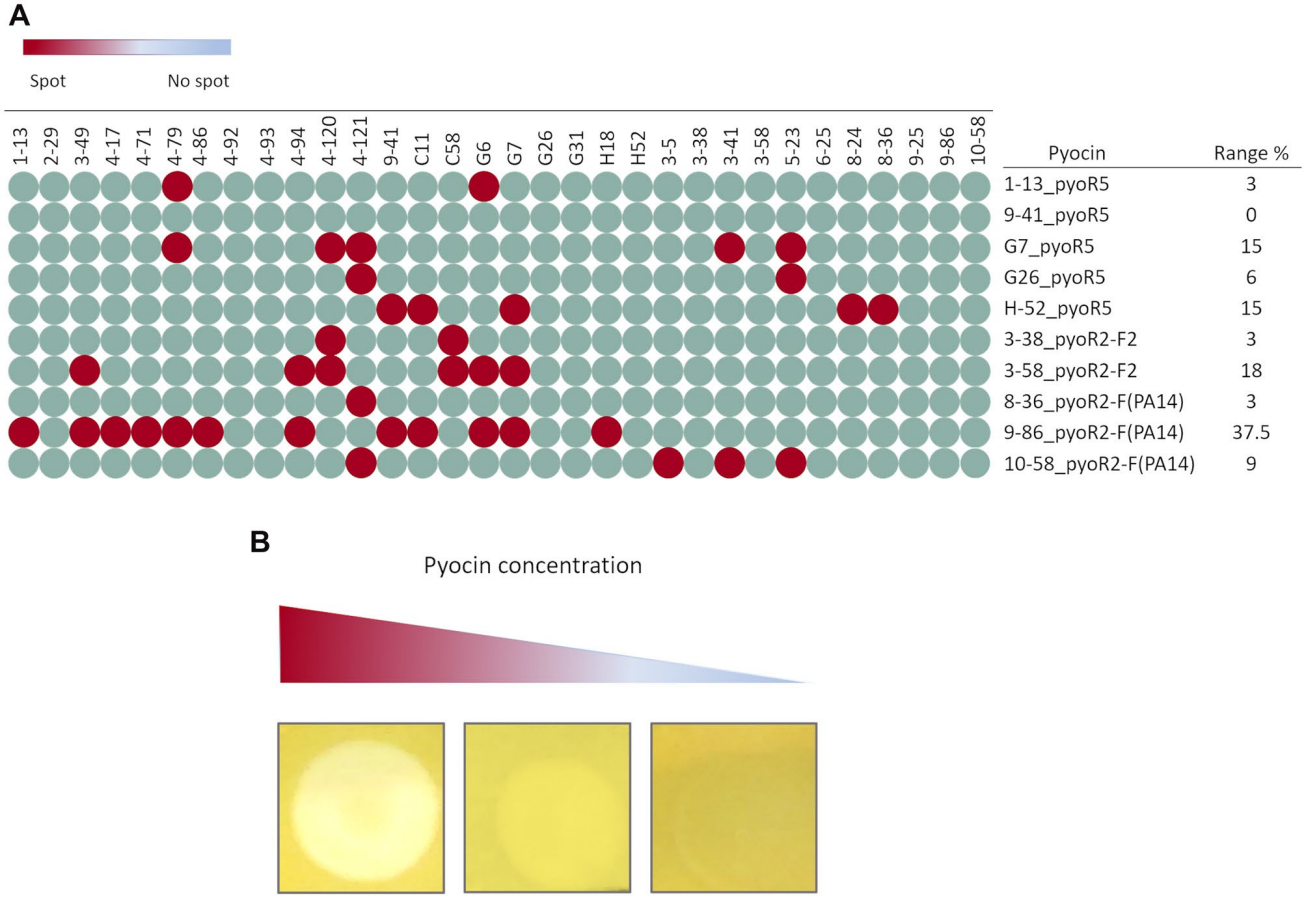
Target range of the pyocins. (**A**) Spot test of the pyocins originating from strains with different ST and serotype. (**B**) Spot production dependent of the pyocin concentration.

The target range of the pyocins was studied by the spot test technique (Fig. 5). The pyocins included in this analysis were selected by according to ST and serotype of the strain from which they were isolated. The results revealed a great variability in the susceptibility of the strains to the pyocins of the same subtype. In addition, no spots occurred when the target strain belonged to the same ST and serotype as the source strain of the pyocin, except for pyocin 10-58_R2-F(PA14), which produced a spot in strain 3–5, which belongs to a different ST but has the same serotype (O11). Furthermore, a variable percentage of target range was observed for all of the pyocins tested, with 9-86_pyoR2F2 showing the highest value and being able to lyse 37.5% of the strains tested.

## Discussion

Pyocins are PTLBs produced by *P. aeruginosa*. Like all PTBLs they are protein complexes with the same structure as phage tails. Like other phage tail particles, such as the type VI secretion systems (T6SS), they also play a role in defence and in interbacterial competition^28^. The genetic and structural similarities between the phage tails and PTBLs initially suggested that the PTBLs evolved as defective phages; however, structural comparison between the T6SS, R-type pyocins and the contractile tail phages suggests evolution from a common ancestor^5,28^.

The presence of pyocins in clinical strains of *P. aeruginosa* seems to be variable. Thus, in a study conducted by Mei et al.^3^, from an analysis of 852 clinical isolates of *P. aeruginosa* they found that 448 belonged to the R-type pyocin and 300 contained genes of the F-type pyocins. From those included in the R-type pyocins 144 belonged to the R1 subtype, 76 to the R2 subtype, 43 to the R5 subtype and the remaining strains were untypeables. Köhler et al.^14^ analysed the tracheal aspirates of 61 patients, in search of R-type pyocins, detecting different R subtype pyocins in 77% of the isolates. In another study of 24 isolates from the lungs of CF patients, all isolates were found to contain R-type pyocins, also mainly of the R1 subtype, and it was concluded that this subtype confers a competitive advantage in biofilms, explaining why certain strains displace others in the CF lungs^6^. In the present study, the genome of 32 clinical isolates of *P. aeruginosa* from several origins, including urinary tract infections, lower respiratory tract infections and intra-abdominal infections, were analysed in search of pyocins, and at least one cluster was found in all isolates. In contrast to the previously mentioned studies, both R-type clusters and F-type clusters were found, resulting in R pyocins and dual R-F pyocins. Analysis of the tail fibers, which determines the specificity (Fig. 1), showed that only R2, R5, F2 and F(PA14) were present in these isolates, with an equal representation of the R5 and R2 subtypes, and a lower representation of F2 than F(PA14) (Fig. 2). In other studies of CF isolates, the R1 subtype was the most commonly identified and in contrast to the results of the present study, R5 was the least well represented subtype^3,6^. In a study conducted by Köhler et al.^14^, pyocins from R1, R2 and R5 were found in the same proportions.

Homology modelling and phylogenetic studies of the pyocins identified in these clinical isolates confirmed the grouping of the pyocins and also revealed the great similarity between them. The main difference observed between the R2-type and the R5-type pyocins was in the genomic region corresponding to the tail fiber, and the same was observed between the F2-type and the F(PA14)-type pyocins (Fig. 3). The tail fiber region has been described as being responsible for the specificity of the pyocins, as it acts as an RBP recognizing the target in the LPS of the target bacteria^5,8^.

Analysis of the pyocins identified in the genomic sequence of the *P. aeruginosa* clinical isolates and the clinical ST, serotype and clinical origin established a relationship between the pyocin type and the ST and serotype, but not the clinical origin of the isolate. Although a relationship with both ST and serotype was identified, it seems that there is more variability between the serotype and the presence of pyocins than with the ST. Thus, the association between the pyocin subtype and the ST and serotype can be extended to the groups established in this work beyond the pyocin subtype, as the A and B groups, which both correspond to an R5-type pyocin, were associated with a different ST and serotype. Although the association between serotype and pyocin subtype has long been recognised^14^, to our knowledge the present is the first report of the relationship with ST. The relationship between the serotype and the pyocin subtype^14^ has always been established with the R-type cluster, but in the present study a relationship between the serotype and the F-type pyocin was demonstrated, as the group C and D pyocins only differ in the F-type cluster. Both groups were related to two different serotypes, but both share O12 and differ in O5 and O11, possibly as a consequence of the presence of the R-pyocin and the F-pyocin, which in this case would confer a competitive advantage to the strain as they are protected by different pyocins^28^. The observed relationship between the serotype, ST and pyocin from the clinical isolates of *P. aeruginosa* tested in this study suggests that the pyocins could potentially be used via analysis of the cluster genomic sequences. The pyocin-serotype association has been used for bacterial typing, but unlike in the present study, the typing was based on the killing activity of a pyocin from an unknown isolate over a collection of indicator strains; this method was abandoned, as it is slow and laborious, and was substituted by molecular methods^17–19^. Currently, thanks to the genome analysis, the study of the PTBLs can complement the traditional typing methods employed in the clinical laboratories.

The serotype in *P. aeruginosa* is determined by the O-antigen, which is a B-band repeating unit of LPS considered a virulence factor^14^. It is known that the pyocin tail fiber proteins recognize the LPS of the competitor strains but do not recognize their own LPS as a target, so they cannot lyse those strains with the same serotype. In a study carried out in 2010, Köhler et al.^14^ deleted different genes responsible for the synthesis of the O-antigen and determined which LPS residues act as receptors for R1-type, R2-type and R5-type pyocins. When the 32 clinical isolates were tested against the 32 isolated pyocins, none were able to lyse the source strain or those strains with the same serotype or ST. As previously described, variability in the susceptibility to the pyocins was observed between those isolates that shared ST and/or serotype and the same type of pyocin, probably due to the frequently observed mutations in the LPS genes in CF, affecting recognition by the RBP^3,29,30^. Pyocins have been considered potential alternatives to antibiotics, and several studies have demonstrated antimicrobial activity of pyocins alone or in combination with other antimicrobials and those sharing the LPS as target^13,22,23,31,32^.

In this study, 32 pyocins belonging to the R5-type, R2-F2 type and R2-F(PA14) type were identified. Homology and phylogenetic analysis established four groups of pyocins (A, B, C and D), each of which was found to be related to the serotype and ST of the source strain. Pyocins are therefore good candidate markers for typing strains of *P. aeruginosa* by analysis of the tail fiber protein of the pyocin. We also observed that they could be used to type R5-type pyocins, by the number and distribution of the genes comprising the pyocin cluster. These pyocins also displayed potential antimicrobial activity as they were able to lyse some of the clinical isolates tested, particularly the 9-86_pyoR2-F(PA14) pyocin, which exhibited the highest range of activity.

## Material and methods

### Strains and culture conditions

Thirty-two clinical strains of *P. aeruginosa* isolated in Portuguese and Spanish hospitals within the framework of two multicentre studies, STEP in Portugal and SUPERIOR in Spain^33^, were used in the study. The software mlst (v2.16.1) (https://github.com/tseemann/mlst) was used for the in silico MLST assignment^33^. Serogroups based on the O-specific antigen (OSA) gene cluster sequences were determined using Blastn tool (v2.9.0+) (http://blast.ncbi.nlm.nih.gov/Blast.cgi) and the OSA database^33^. The origin, ST and serotype of the isolates are shown in Table 1. All strains were grown in Luria–Bertani broth (LB) medium (0.5% NaCl, 0.5% yeast extract, 1% tryptone) at 37 °C and 180 rpm. LB was supplemented with agar (1.5%) when necessary.

### Identification of pyocin clusters in silico

The genomes of 32 clinical isolates *P. aeruginosa* (NCBI BioProject: PRJNA629475) were analysed to search for pyocins. For this purpose, the anthranilate flanking genes, *trpD* and *trpE*, were identified in the search for the genes that typically compose the pyocin clusters, which have been reported to be included between these genes^5^.

When the pyocin cluster sequences were distributed in several contigs, they were then compared by homology and assembled using BLASTn and Vector NTI Advance™ 11 (Invitrogen) programs. The complete sequences of the pyocin clusters were annotated by RAST^34^, HMMER (http://hmmer.org) and HHPRED^35^. The pyocin cluster R2-F2 of *P. aeruginosa* PAO1 was used as a reference sequence, which corresponds to the region between the genes PAO610-PAO648 (Genbank: AE004091.2-AE004091.2) from the *Pseudomonas aeruginosa* database^36^.

All the nucleotide sequence data reported are available in the Third Party Annotation Section of the DDBJ/ENA/GenBank databases under the accession numbers TPA: BK062614–BK062645 (Table 2).

### Pyocin cluster type identification

The pyocin cluster types were assigned by homology with the tail fiber corresponding to the protein of the reference strain *P. aeruginosa* PAO1: PAO620 for the R-type pyocins and by PAO646 for the F-type pyocins^12^.

These strains were compared by BLASTp against the corresponding protein of the different pyocin types: R1 (ARI05994.1), R2 (AAG04009.1), R3 (ABP93392.1), R4 (ABP93394.1), R5 (ABP93396.1), F2 (AAG04035.1) and F(PA14) (ABJ15607.1). The pyocin clusters were considered to belong to a subtype when the homology value was higher than 90%.

### Homology and phylogenetic analysis

The sequences obtained for the different pyocin clusters were analysed in order to study their homology. The studies were carried out using the Easyfig 2.2.5^37^ and BRIG 0.95^38^ tools with the tBLASTx option. The Query Cover and Identity values were analysed with BLASTn.

The sequences were aligned and a phylogenetic tree was constructed with the bioinformatic software Geneious Prime (Dotmatics).

### Extraction and concentration of phage tail-like bacteriocins

The selected strains used as sources of pyocins were cultured overnight in LB broth at 37 °C. The culture was then diluted 1:100 in LB and incubated at 37 °C and 180 rpm. Once the optical density measured at a wavelength of 600 nm (OD_600nm_) of 0.4 was reached, 10 µg/ml of mitomycin C (Sigma-Aldrich) was added and the culture was incubated until it turned clear. The lysed cultures were centrifuged at 4000 rpm for 10 min, and the supernatant with the pyocins was recovered and incubated with 1% chloroform for 30 min. Finally, the supernatant with the pyocins was filtered through a 45 µm filter (FILTER-LAB®PES Syringe filter).

For concentration, the pyocins were precipitated with polyethylene glycol (PEG). The pyocin solution was precipitated overnight at 4 °C with 10% PEG and 0.5 M NaCl. The pyocins were collected by centrifugation for 15 min at 11,000 rpm and 4 °C. The supernatant was discarded and the pellet suspended in SM buffer (0.1 M NaCl, 1 mM MgSO4, 0.2 M Tris–HCl, pH 7.5), to obtain a tenfold concentration. Finally, a 1:1 volume of chloroform was added and incubated with gentle shaking for 20 min, and the phases were then separated by centrifugation for 10 min at 4000 rpm. The aqueous phase with the pyocin suspension was recovered and stored at 4 °C until use.

### Pyocin transmission electron microscopy (TEM)

The pyocin solutions were fixed in a grid and negatively stained in 1% aqueous uranil acetate for 5 min and examined in a transmission electron microscope JEOL JEM-1011.

### Pyocin killing spectrum

In order to determine the target range of each isolated pyocin, each was tested against the 32 strains from which they were isolated (Table 1). The killing activity was assayed by spot test^39^. Briefly, double agar layer plates were prepared with the putative host strain mixed with the soft upper agar layer (0.4% agar). Once solidified, a drop (10 μl) of the pyocin solution was deposited on top of the agar layer, and the plates were incubated at 37 °C for 20 h.

To differentiate the R-type and F-type pyocins from the S-type pyocins, proteinase K was added to the plates, as R and F-type pyocins are protease resistant and S-type is protease sensitive^12^. In order to differentiate the pyocins from prophages induced with mitomycin C, serial dilutions were spotted on agar plates. When no individual plaques were observed at the higher dilutions, the presence of a spot was considered to be the result of the killing activity of the pyocin.

## Supplementary Information

Supplementary Table S1.

## Data Availability

All data generated or analysed during this study are included in this published article [and its supplementary information files].
